# DNA damage induced by Strontium-90 exposure at low concentrations in mesenchymal stromal cells: the functional consequences

**DOI:** 10.1038/srep41580

**Published:** 2017-01-30

**Authors:** S. Musilli, N. Nicolas, Z. El Ali, P. Orellana-Moreno, C. Grand, K. Tack, S. Kerdine-Römer, J. M. Bertho

**Affiliations:** 1 Institut de radioprotection et de sureté nucléaire (IRSN), PRP-HOM/SRBE/LRTOX, Fontenay aux roses, France; 2 UMR996 - Inflammation, Chemokines and Immunopathology, INSERM, Université Paris-Sud, Université Paris-Saclay, 92296, Châtenay-Malabry, France

## Abstract

^90^Sr is one of the radionuclides released after nuclear accidents that can significantly impact human health in the long term. ^90^Sr accumulates mostly in the bones of exposed populations. Previous research has shown that exposure induces changes in bone physiology both in humans and in mice. We hypothesize that, due to its close location with bone marrow stromal cells (BMSCs), ^90^Sr could induce functional damage to stromal cells that may explain these biological effects due to chronic exposure to ^90^Sr. The aim of this work was to verify this hypothesis through the use of an *in vitro* model of MS5 stromal cell lines exposed to 1 and 10 kBq.mL^−1^ of ^90^Sr. Results indicated that a 30-minute exposure to ^90^Sr induced double strand breaks in DNA, followed by DNA repair, senescence and differentiation. After 7 days of exposure, MS5 cells showed a decreased ability to proliferate, changes in cytokine expression, and changes in their ability to support hematopoietic progenitor proliferation and differentiation. These results demonstrate that chronic exposure to a low concentration of ^90^Sr can induce functional changes in BMSCs that in turn may explain the health effects observed in following chronic ^90^Sr exposure.

Strontium-90 (^90^Sr) is a bone- and teeth-seeking radionuclide^1^ that is released in large quantities during nuclear accidents and aerial nuclear testing^2^,^3^,^4^. Due to its high solubility in water and long half-life (29 years), this radionuclide persists in the environment for a long time and progressively enters the food chain. As a consequence, some human populations are exposed to ^90^Sr through ingestion over the long term. For instance, the Techa River was heavily contaminated during the 1950 s, resulting in the exposure of people living by the riverside^5^. Studies on the Techa River cohort demonstrated that some patients presented symptoms of chronic radiation syndrome (CRS) with a suppression of hematopoiesis and immune defense^6^,^7^. A decreased bone remodeling rate was also observed in this population^8^. In addition, we demonstrated an increased bone resorption^9^ and a reduced immune response to a vaccine challenge^10^ in mice exposed to ^90^Sr through ingestion for 20 weeks. However, the mechanisms underlying these health effects remain unclear.

Stable strontium is considered as a low toxicity element with a non-observed adverse effect level (NOAEL) in mice of between 40 and 500 mg/kg bw/day according to the physiological system studied^11^. In addition, *in vitro* models of strontium activity on osteoblasts showed no effects caused by this element at concentrations less than 1 × 10^−3^ M^12^ or l ×10^5^ M^13^ depending on the model. We thus hypothesize that the potential effect due to ^90^Sr might be linked to irradiation due to its disintegration. In line with this hypothesis, the β rays emitted by ^90^Sr are of high energy (0.54 and 2.26 MeV), with a mean penetration range of 150–200 μm in living tissue. As a consequence, the energy of ionizing radiations is deposited in small volumes around the bone tissue, and especially in the endosteum and in cells lining the bone tissue, i.e. bone marrow stromal cells (BMSCs) and hematopoietic stem cells (HSCs)^14^. Mesenchymal stem cells (MSCs), key partners of the HSC niche, are known to play a central role in the maintenance of HSC stemness and have been demonstrated to support hematopoiesis^15^ through the expression of numerous growth factors and adhesion molecules^16^. MSCs can also differentiate into several lineages including adipocytic and osteoblastic lineages, which also play a role in bone physiology^17^.

In order to verify this hypothesis, we modeled such an exposure through the use of BMSC and MS5 cell lines cultured in the presence of 1 or 10 kBq.mL^−1^ of ^90^Sr. The lowest concentration used was close to the one found in mice bones after 24 weeks of chronic ^90^Sr ingestion^1^, taking into account the geometric analogy between the bone and bone marrow tissues. This model was then used to assess functional damage induced by ^90^Sr in BMSCs. We showed in this model that ^90^Sr at low concentrations is able to induce DNA damage, senescence and differentiation in stromal cells, which in turn induces phenotypic and functional changes.

## Results

### ^90^SrCl_2_ exposure at low concentration induces double strand-breaks (DSB) in BMSCs

**Figure 1:**
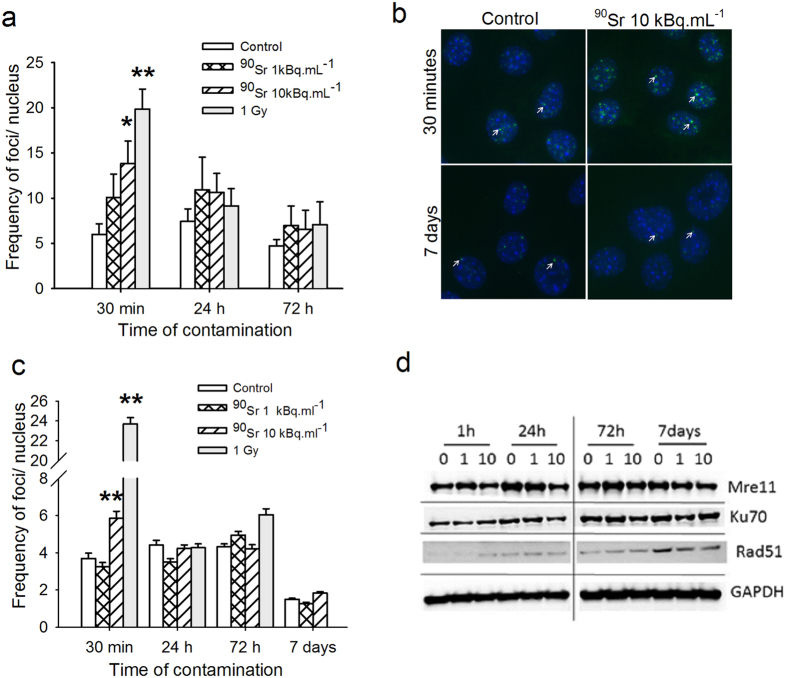
^90^Sr-induced double-strand breaks and DNA repair. (**a**) Quantification of γ-H2AX foci per nucleus in rat BMSCs after 30 minutes, 24 and 72 hours of exposure to ^90^Sr or external 1 Gy γ-irradiation used as a positive control (Student’s t-test, *p < 0.05 and **p < 0.001, n = 3). (**b**) Representative immunostaining of γ-H2AX in MS5 control cells and after 30 minutes (upper panel) or 7 days (lower panel) of exposure to 10 kBq.ml^−1^ of ^90^Sr. γ-H2AX foci are stained in green and are indicated by the arrows, nuclei are stained in blue. (**c**) Quantification of γ-H2AX foci per nucleus in MS5 cells after 30 minutes, 24 hours, 72 hours and 7 days of exposure to ^90^Sr or external 1 Gy γ-irradiation as positive control (ANOVA on Ranks, H = 372.996, dof = 3, **p < 0.001). (**d**) Representative images of Western blot for Mre11, Ku70 and Rad51 proteins, from 1 hour to 7 days of exposure. GAPDH represents the loading control. Abbreviations: 0: Control cells; 1: ^90^Sr 1 kBq.mL^−1^; 10: ^90^Sr 10 kBq.mL^−1^. Full length blots uncorrected for contrast are presented in Supplementary Figure 1.

Immunostaining of γ-H2AX foci in rat BMSCs was carried out in order to assess if ^90^Sr exposure at low concentrations is able to induce DSB in cell DNA^18^. Flow cytometry analysis of rat BMSCs showed that cells were 82.2 ± 9.2% CD73^+^ CD90^+^ and 79.6 ± 10.4% CD29^+^, a feature of rat mesenchymal stromal cells^19^. It is well known that cells form DSB during the S-phase due to the replicative forks. In fact, the frequency of DSB in log phase cell cultures showed a high background of γ-H2AX foci, making it impossible to detect a potential increase in DSB due to ^90^Sr exposure (data not shown). Consequently, all of our experiments were performed after cells reached confluence in order to limit such an effect. ^90^Sr exposure induced a significant increase in the frequency of γ-H2AX foci per nucleus just 30 minutes post-exposure to 10 kBq.mL^−1^ of ^90^Sr but not to 1 kBq.mL^−1^ (Fig. 1a, 5.98 ± 1.17 foci per nucleus in control cells and 13.82 ± 2.49 foci per nucleus in 10 kBq.mL^−1^ exposed cells, Student’s *t*-test, p = 0.012). By comparison, a 1 Gy external exposure with gamma rays induced the appearance of 23.6 ± 0.7 γ-H2AX foci per cell. Thus, the exposure of MSCs to ^90^Sr induced a two-fold smaller increase in γ-H2AX foci compared to the induction of γ-H2AX foci by 1 Gy external exposure. However, the background level of γ-H2AX foci was high in these cells, probably due to a large heterogeneity in cell cycle status of these primary BMSC cultures. To confirm these results, the MS5 cell line was used throughout the present study. Similarly, the results showed a significant higher number of γ-H2AX foci per nucleus in MS5 cells after 30 minutes of exposure to 10 kBq.mL^−1^ of ^90^Sr as compared to the negative control (Fig. 1b and c, 5.84 ± 0.38 and 3.69 ± 0.29 foci per nucleus respectively, one-way ANOVA, p < 0.001). By contrast, no changes were observed with 1 kBq.mL^−1^. As compared to the external exposure to 1 Gy of gamma rays, exposure of MS5 cells to 10 KBq.ml^−1^ of ^90^Sr induced a 10% increase in DSB. Additionally, a rapid decrease in the number of γ -H2AX foci per nucleus was seen after 24 hours, 72 hours and 7 days of contamination, returning to control levels. These kinetics are similar to those obtained after 1 Gy of external γ-irradiation used as a positive control in both BMSCs and MS5 (grey bars in Fig. 1a and c), and suggest that DNA damage is rapidly induced by exposure to ^90^Sr, but that DNA repair mechanisms are also rapidly activated.

The level of proteins involved in the DNA repair pathway (Mre11, Ku70 and Rad51) was thus analyzed after 1 hour, 24 hours, 72 hours and 7 days of exposure to ^90^Sr (Fig. 1d). In control cells these proteins are already highly expressed. The quantification of bands (data not shown) indicated that there was a slight increase for Ku70, Mre11 and Rad51 protein expression in exposed groups after 1 hour. After 7 days of contamination, this expression returned to basal level. These results suggested that MS5 cells are able to repair the double strand breaks induced by the ^90^Sr contamination, with a similar kinetics as with DSB induced by external irradiation.

### Redox status of MS5 cells is not affected by ^90^SrCl_2_ exposure

**Figure 2:**
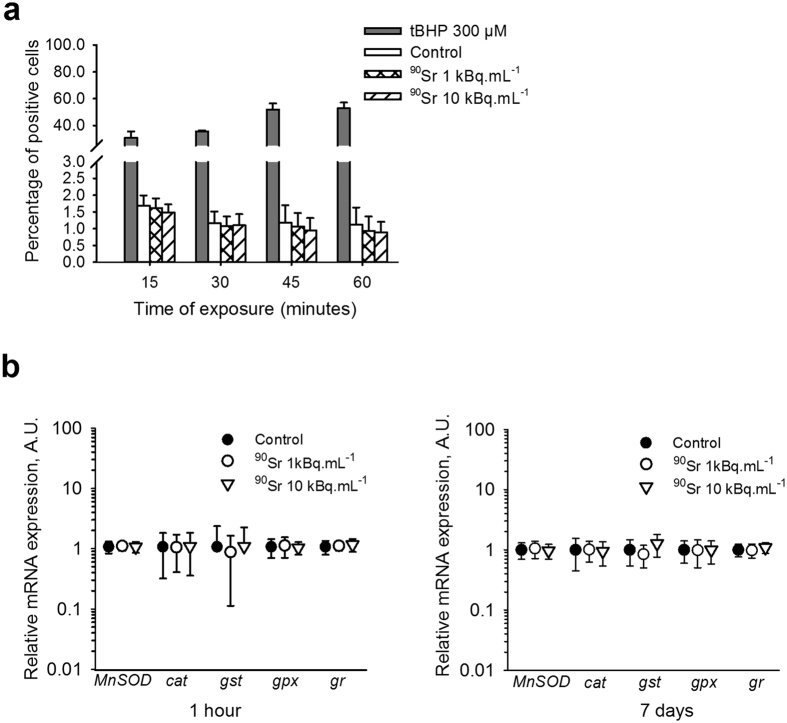
Oxidative stress is not induced by ^90^Sr exposure. (**a**) Flow cytometry detection of reactive oxygen species production after 15 minutes to 1 hour of ^90^Sr exposure. Grey bars represent cells treated with 300 μM tBHP as positive control (two-way ANOVA, F_(2. 60)_ = 0.854, n.s.). (**b**) Relative mRNA expression of the major enzymes involved in the anti-oxidative system: *MnSOD, Cat, Gst, Gpx* and *Gsr* after 1 hour (left panel) and 7 days (right panel) of exposure to ^90^Sr (one-way ANOVA, n.s.). Results are presented as mean ± standard deviation (SD) (arbitrary units, A.U.).

Reactive oxygen species (ROS) production is known to be one of the earliest consequences of ionizing radiation and can contribute to DSB formation^20^,^21^. The ability of ^90^Sr to induce oxidative stress in MS5 cells was thus evaluated. First, the general ROS production was measured using a fluorescent reporter probe, H2DCFDA. tBHP as an H_2_O_2_ provider was used as a positive control and showed the induction of a high level of oxidative stress in MS5 cells, reaching a peak after only 45 minutes of exposure. By contrast, ^90^Sr exposure did not induce a significant increase in oxidative stress regardless of ^90^Sr concentration and the time point (Fig. 2a). When focusing on the gene expression of the main enzymes involved in the antioxidant system such as catalase (*Cat*), manganese superoxide dismutase (*MnSOD*), glutathione-S-transferase (*Gst*), glutathione peroxidase (*Gpx*), and glutathione reductase (*Gsr*) between 1 hour and 7 days of ^90^Sr contamination, no significant modification in the transcription of these genes was observed between groups (Fig. 2b). This indicates that, at these concentrations, ^90^Sr is not able to induce a significant increase in the redox status level which could affect MS5 cells. This is consistent with the direct proportionality of oxidative stress induction with energy deposition by ionizing radiation and with the low dose rate in our experiments, calculated at 6.5 mGy.h^−1^ (A. Desbrée, personal communication).

### ^90^SrCl_2_ exposure induces senescence in MS5 cells

**Figure 3:**
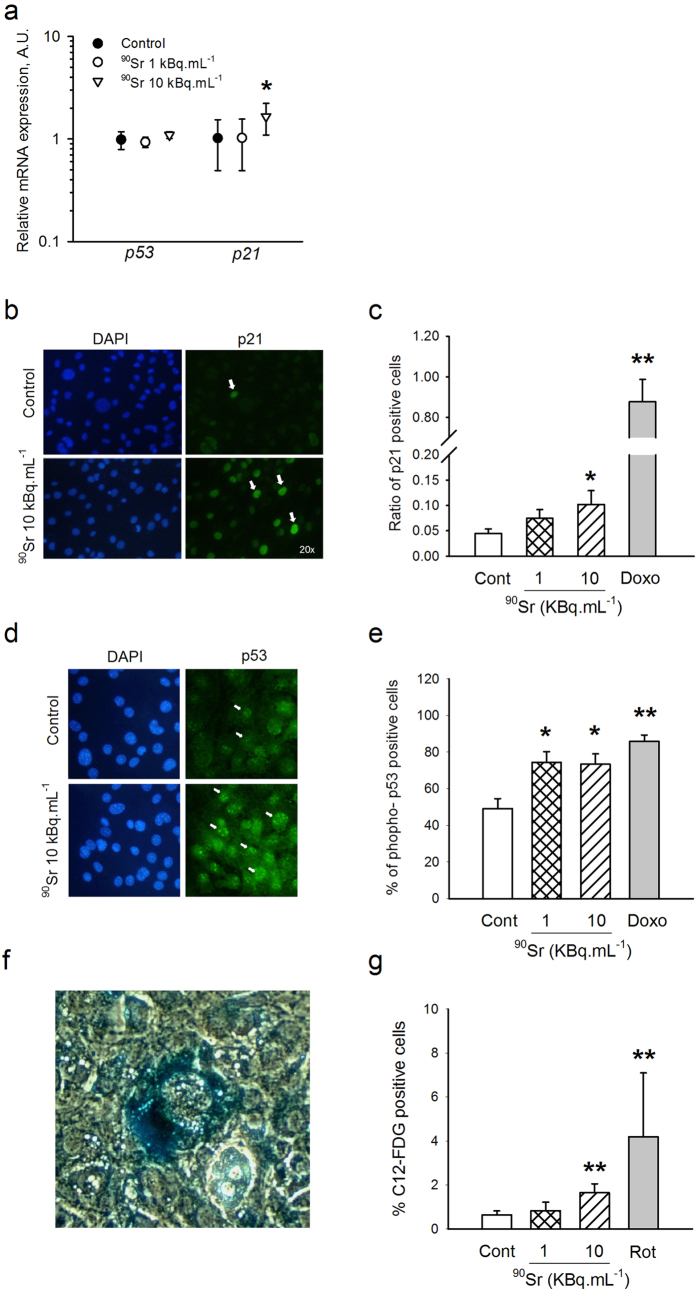
Senescence induction in MS5 cells by ^90^Sr exposure. (**a**) Relative mRNA expression of *p21* and *p53* genes in MS-5 cells after one week of contamination by ^90^Sr (one-way ANOVA, F_(3, 28)_ = 3.12, *p < 0.05). Results are presented as mean ± standard deviation (SD) (arbitrary units, A.U.). (**b**) Representative immunostaining of p21 in MS5 control cells (upper panel) and after 7 days of exposure to 10 kBq.mL^−1^ of ^90^Sr (lower panel). p21 positive cells are stained in green and are indicated by white arrows, nuclei in blue. (**c**) Ratio of p21 positive cells after 7 days of ^90^Sr exposure or after 6 hours of treatment with 2 μM doxorubicin used as positive control (Student’s t-test, n = 3, *p < 0.05 and **p < 0.001). **(d**) Representative immunostaining of p53 in MS5 control cells (upper panel) and after 1 hour of exposure to 10 kBq.mL^−1^ of ^90^Sr (lower panel). p53 positive cells are stained in green and are indicated by white arrows, nuclei in blue. (**e**) Percentage of p53 positive cells after 1 hour of ^90^Sr exposure or after treatment with 2 μM doxorubicin used as positive control (Student’s t-test, n = 3, *p < 0.05 and **p < 0.001). (**f**) A positive cell for SA-β-Gal staining showing enlarged nucleus and cytoplasm compared to surrounding cells. (**g**) Flow cytometry detection of SA-β-galactosidase activity after 7 days of culture with either ^90^Sr exposure during the whole culture period or 24 hours stimulation with 0.1 μM rotenone used as a positive control. Results are expressed as percent of positive cells (Student’s t-test, n = 10, **p < 0.001).

A possible consequence of DNA damage and repair is senescence, as previously described after low-dose irradiation^22^,^23^. The senescent status of MS5 cells was first assessed by studying *p21* gene expression (Fig. 3a), a well-known marker of cellular senescence^24^. A significant increase in relative *p21* expression compared to the negative control was seen in MS5 cells after 6 hours of treatment with 2 μM doxorubicin as a positive control (5.71 ± 0.53 fold increase compared to the control, Student’s t-test, p < 0.001, data not shown). A similar but lower pattern is observed in MS5 cells exposed for one week to 10 kBq.mL^−1^ of ^90^Sr (1.65 ± 0.13 fold increase, one-way ANOVA, p = 0.03) but not with 1 kBq.mL^−1^ (Fig. 3a). This pattern of senescent cells was confirmed by the immunostaining of *p21* after 6 hours of treatment with doxorubicin or after one week of continuous exposure to ^90^Sr, where a dose-dependent increase of the percentage of *p21*-positive cells was observed (Fig. 3b and c). This increase was significant for the 10 kBq.mL^−1^ contaminated group compared to the control (Fig. 3c, 10.2 ± 2.7% and 4.46 ± 0.91% of positive cells respectively, Student’s t-test, p = 0.015) but not for the 1 kBq.mL^−1^ exposure (Fig. 3c). Again, this response was of low amplitude, representing roughly 5% of the increase observed in the positive control. It has previously been shown that the activation of *p21* is controlled by *p53*^25^. No change in *p53* gene expression levels was observed after ^90^Sr exposure (Fig. 3a). However, immunostaining experiments revealed a significant increase in the phosphorylation of *p53* after 1 hour of treatment either with 2 μM doxorubicin or with ^90^Sr exposure at 1 and 10 kBq.mL^−1^, as compared to the negative control (Fig. 3d and e, respectively 85.8 ± 3.47%. 74.7 ± 5.93% and 73.4 ± 5.62% of *p53* positive cells, Student’s t-test p < 0.05 and p < 0.001). In order to confirm the increased senescent status of MS5 cells after one week’s exposure to ^90^Sr, cells were tested for senescence-activated β-galactosidase (SA-β-Gal). Cells positive for SA-β-Gal activity presented a typical morphology of senescent cells, with an enlarged cytoplasm and nucleus (Fig. 3f). In order to obtain a more accurate evaluation of senescence induction, SA-β-Gal positive cells were quantified using 5-dodecanoylaminofluorescein di-b-D galactopyranoside (C_12_-FDG) as a substrate for SA-β-gal and rotenone as a senescence-inducing positive control^26^. Results clearly indicated a slight but significant increase in SA-β-Gal positive cells in MS5 cells exposed to 10 KBq.ml^−1^ of ^90^Sr, but not in cells exposed to 1 KBq.ml^−1^, as compared to the negative control (Fig. 3g). Furthermore, a significant increase in mean fluorescence intensity was observed in MS5 cells exposed to 10 KBq.ml^−1^ of ^90^Sr, but not in cells exposed to 1 KBq.ml^−1^, in comparison to the negative control (data not shown). Again, the response of MS5 cells to ^90^Sr was modest, representing 40–50% of the response observed with rotenone. Overall, these results suggested that the highest concentration of ^90^Sr used in this study was able to induce cellular senescence during the 7 days of exposure, through the *p53*/*p21* pathway^25^.

### ^90^SrCl_2_ exposure at low concentrations affects cell proliferation but not cell death

**Figure 4:**
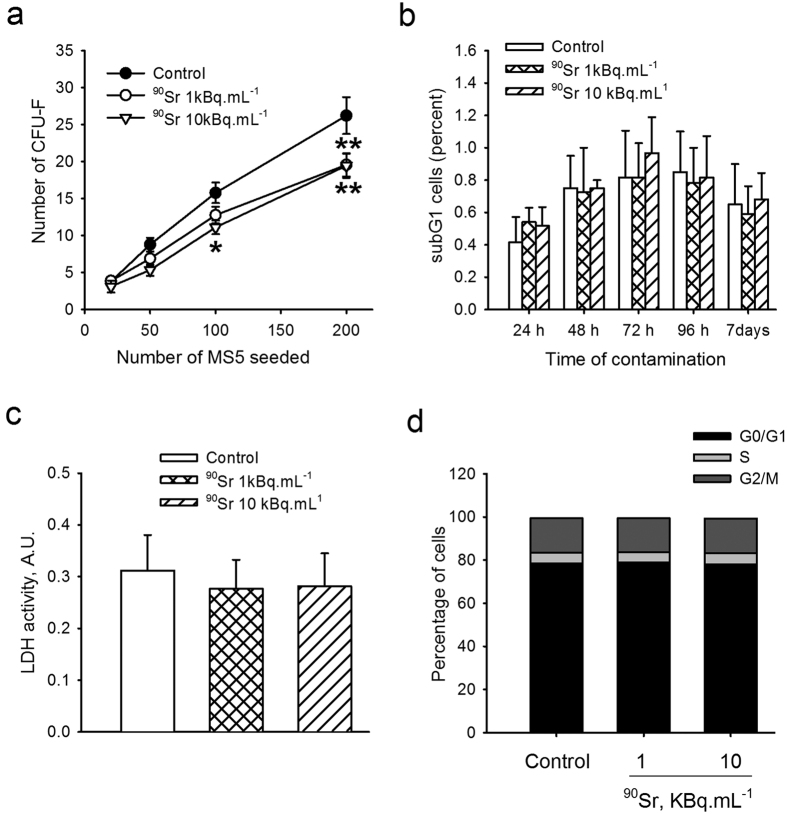
^90^Sr exposure decreases cell proliferation without increasing cell death. (**a**) Results of CFU-F assay after ^90^Sr exposure (two-way ANOVA, F_(2, 96)_ = 10.38, p < 0.001). Differences between groups were signified for *p < 0.05 and **p < 0.001. (**b**) Percentage of MS5 cells in subG1 phase from 24 hours to 7 days of contamination (two-way ANOVA. F_(2, 80)_ = 0.214, n.s.). (**c**) Extracellular LDH activity (arbitrary units, A.U.) in cell supernatant after 7 days of exposure (one-way ANOVA, n.s.). (**d**) Percentage of MS-5 cells in G0/G1, S or G2/M phases after 7 days of exposure to ^90^Sr (one-way ANOVA, n.s.).

Senescence is also defined by an arrest of cell proliferation^27^. The proliferation ability of MS5 after exposure to ^90^Sr was assessed using the CFU-F assay as originally described^28^. MS5 cells were exposed to ^90^Sr for one week after they reached confluence and were then tested for their ability to form CFU-F. A decrease in the number of CFU-F after exposure was observed with 10 kBq.mL^−1^ of ^90^Sr compared to the control (Fig. 4a, two-way ANOVA, p < 0.001). A slight but non-significant decrease in CFU-F numbers was also observed with 1 kBq.mL^−1^ of ^90^Sr. This decrease in CFU-F frequency demonstrated a decreasing proliferation ability of MS5 cells after exposure to ^90^Sr. It should be noted that a similar decrease in CFU-F numbers (although at a lesser magnitude) was observed with MS5 cells exposed to ^90^Sr and tested for CFU-F ability in the log phase of the culture (data not shown).

In order to determine whether the decrease in cell proliferation was due to an increase in cell death, the subG1 fraction of cells was analyzed after ^90^Sr exposure. Results showed no difference in the percentage of MS5 cells in subG1 phase for both ^90^Sr concentrations and for each time point (Fig. 4b). Furthermore, the measurement of LDH activity (used as a marker of cytotoxicity) did not show any difference between exposure groups and the control group (Fig. 4c). This indicated that exposure to ^90^Sr at these concentrations did not induce significant cell death.

Since senescent cells are known to be in a state of proliferative arrest, the impact of ^90^Sr contamination on cell cycle phases by PI staining was tested. The results demonstrated that cells progressed in their cell cycle between 24 hours (not shown) and 7 days of exposure (Fig. 4d), with an increase in the proportion of cells in G0/G1 and this despite ^90^Sr exposure. However, no significant changes in the relative proportion of cells in each phase of the cell cycle were observed regardless of the ^90^Sr concentration and the time point tested. These results suggest that these concentrations of ^90^Sr were not able to induce a significant cell cycle arrest.

### MS5 cell differentiation was increased by ^90^SrCl_2_ exposure

**Figure 5:**
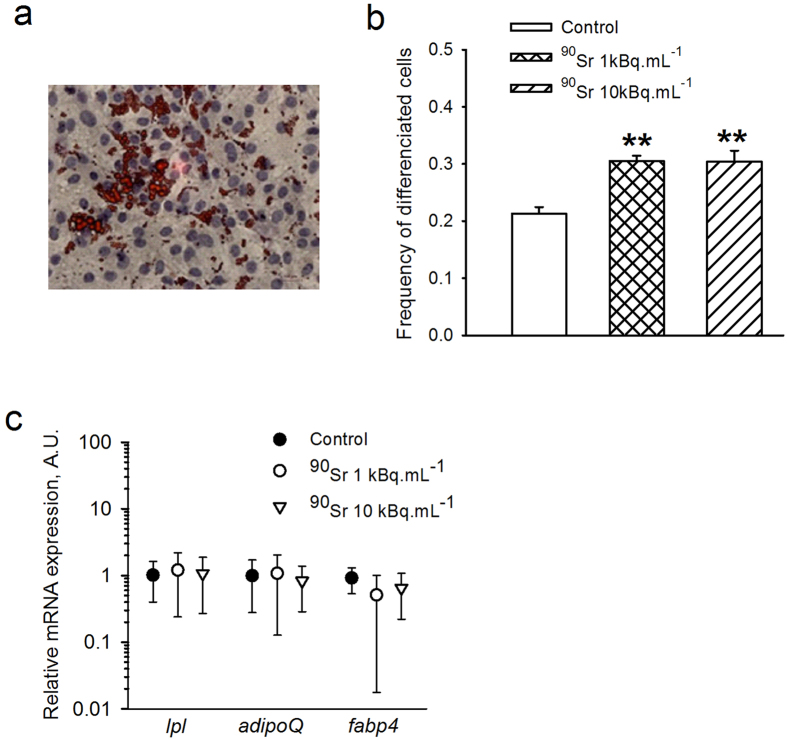
^90^Sr exposure induces adipocytic differentiation. (**a**) Representative image of Oil Red O staining of MS5 cells. Lipid droplets are stained in red, nuclei in blue. (**b**) Frequency of differentiated cells after one week of exposure to ^90^Sr (one-way ANOVA, F_(3, 68)_ = 9.251, **p < 0.001). (**c**) Relative mRNA expression of the main adipocyte markers: *Lpl, Adipoq* and *Fabp4* in MS5 after one week of contamination with ^90^Sr (one-way ANOVA, n.s.). Results are presented as mean ± standard deviation (SD) (arbitrary units, A.U.).

MS5 cells belong to the mesenchymal lineage but retain the characteristics of pre-adipocytic cells^29^. This feature was used to determine the impact of ^90^Sr contamination on the spontaneous ability of MS5 cells to differentiate into adipocytes. This was first assessed by histological staining of lipid vesicles (Fig. 5a). The frequency of adipocytic cells revealed a significant higher rate of differentiation after one week’s exposure to both 1 and 10 kBq.mL^−1^ as compared to control cells (Fig. 5b, 0.305 ± 0.009 and 0.304 ± 0.019 *versus* 0.213 ± 0.011 respectively, one-way ANOVA, p < 0.001). The gene expression of adipogenic markers such as *Lpl, Adipoq* and *Fabp4* was also analyzed after 7 days of exposure to ^90^Sr (Fig. 5c), but no changes in these markers’ expression levels were seen in any group. This is probably due to the high level of expression of these genes due to the pre-adipocyte status of MS5 cells^29^.

### Sustainment of hematopoietic cell differentiation by MS5 cells after exposure to ^90^SrCl_2_

**Figure 6:**
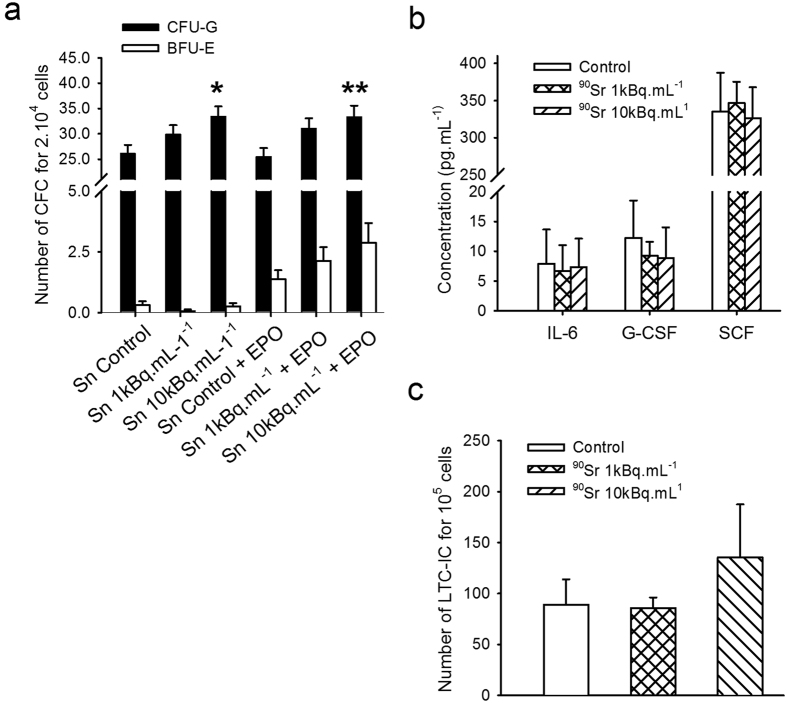
^90^Sr exposure modifies cellular functions. (**a)** Number of CFCs per 20,000 cells after 12 days of culture with conditioned medium in methylcellulose medium without growth factors (one-way ANOVA, F_(4, 65)_ = 2.523, *p < 0.05) or supplemented with erythropoietin ( + EPO, one-way ANOVA, F_(4, 65)_ = 3.685, **p < 0.001). Abbreviations: Sn, Supernatant; EPO: Erythropoietin. (**b**) Concentration of IL-6, SCF and G-CSF (pg.mL^−1^) in cell supernatants after 7 days of exposure to ^90^Sr (one-way ANOVA, n.s.). GM-CSF and IL-3 concentrations were below the detection limit. (**c**) Number of CFCs per 10^5^ cells obtained by the LTC-IC assay (one-way ANOVA, n.s.).

One of the main roles of MSCs is the sustainment of hematopoietic cell proliferation and differentiation. In fact, the MS5 cell line is frequently used as a feeder layer in the long term culture initiating cell (LTC-IC) assay^30^. In order to define the potential impact of ^90^Sr exposure on MS5 functions, supernatants from MS5 cells exposed to ^90^Sr for one week or from control cultures were used to supplement methylcellulose medium (at a 10% concentration) in a colony forming cell (CFC) assay. As a control medium, supernatant from control cultures of MS5 were supplemented with 10 kBq.mL^−1^ of ^90^Sr in order to take into account a possible direct effect of ^90^Sr on bone marrow progenitor proliferation. Results (Fig. 6a) showed a significant increase in CFU-G frequency when bone marrow cells were grown in the presence of supernatant from 10 kBq.mL^−1^ exposed MS5 cells as compared to a control supernatant (33.4 ± 1.96 CFU-G for 2 × 10^4^ cells and 26.1 ± 1.7 CFU-G for 2 × 10^4^ cells respectively, one-way ANOVA, p = 0.003). A similar significant increase in the frequency of CFU-G but not in the frequency of BFU-E exposed to supernatant from 10 kBq.mL^−1^ of ^90^Sr exposed MS5 was observed with a methylcellulose medium supplemented with erythropoietin (33.3 ± 2.2 CFU-G for 2 × 10^4^ cells and 25.5 CFU-G for 2 × 10^4^ cells respectively, one-way ANOVA, p < 0.001). By contrast, the use of supernatant from MS5 exposed to 1 kBq.mL^−1^ of ^90^Sr, or exposed to control supernatant supplemented with 10 kBq.mL^−1^ of ^90^Sr, did not induce a change in both CFU-G and BFU-E frequencies (data not shown). This result suggests that the exposure of MS5 cells induced some changes in the cytokine release by these cells. Consequently, we tested the extracellular production of IL-3, IL-6, SCF, GM-CSF and G-CSF, which are some of the main cytokines involved in progenitor proliferation (Fig. 6b). No significant differences in IL-6, SCF and G-CSF release were observed between groups after one week of exposure to ^90^Sr, and the concentrations of IL-3 and GM-CSF in these MS5 supernatants were below the detection limit.

To go further, the sustainment role of MS5 was investigated through the LTC-IC assay, in which mouse Lin^-^ bone marrow cells were cultured for 5 weeks in the presence of MS5 cells either previously exposed to ^90^Sr or not, before being tested for their ability to form CFCs^30^,^31^. The frequency of LTC-ICs in control cultures was 88.5 ± 55.6 per 1 × 10^5^ Lin^−^ bone marrow cells and was not significantly affected by the pre-exposure of MS5 cells to ^90^Sr regardless of the concentration used (Fig. 6c).

### Cytokine release after ^90^Sr exposure

**Figure 7:**
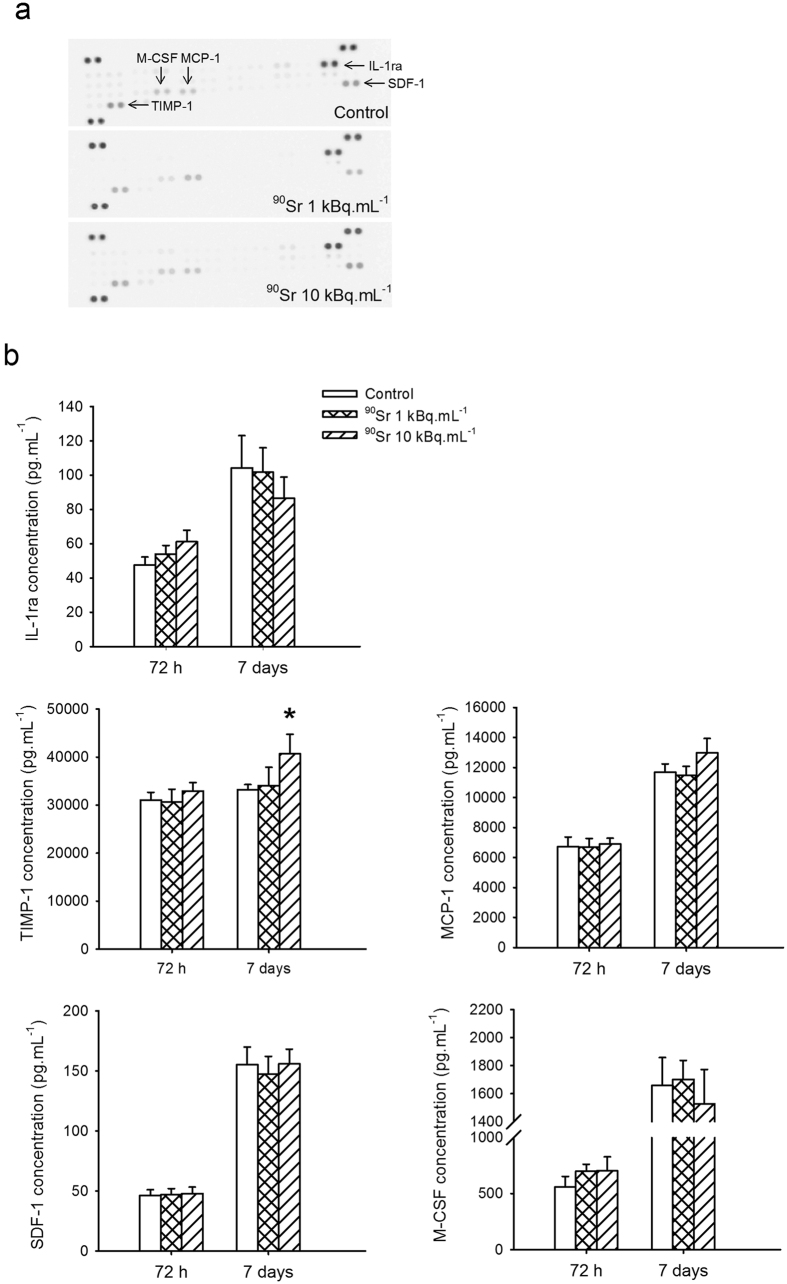
^90^Sr exposure and cytokine release. (**a**) Intracellular cytokine levels obtained by cytokine array in control cells or after 7 days of exposure to 1 or 10 kBq.mL^−1^ of ^90^Sr. Results are presented as uncropped blots, uniformly adjusted for contrast. (**b**) Concentrations of IL-1ra, M-CSF, TIMP-1, MCP-1 and SDF-1 (pg.mL^−1^) in cell supernatants in control cells and after 72 hours and 7 days of exposure to 1 and 10 kBq.mL^−1^ of ^90^Sr (Student’s t-test, n = 4, *p < 0.05).

The previous results suggested that exposure of MS5 cells to ^90^Sr is able to induce some changes to the secreted factors, either quantitatively or qualitatively, but do not seem to have an effect on cell-cell contact, i.e. on the expression of adhesion molecules at the cell surface of MS5 cells. In order to analyze the possible changes in a broader way, the intracellular level of cytokines in MS5 after 7 days of exposure to ^90^Sr was then screened using a protein array (Fig. 7a). The main cytokines identified in the cytoplasm of MS5 cells after 7 days in any exposure group were IL-1ra, M-CSF, MCP-1, TIMP-1 and SDF-1. When measuring these cytokines in the culture supernatant, no significant changes were observed between exposure groups at 72 hours (Fig. 7b). After 7 days of exposure, a slight significant increase in TIMP-1 concentration was observed in the ^90^Sr 10 kBq.mL^−1^ contaminated cells compared to control (40,680.7 ± 4,059.4 and 33,199.5 ± 1,070.9 pg.mL^−1^ respectively, Student’s t-test, p = 0.038). No other significant changes were observed despite a tendency IL-1ra concentration and an increase in MCP-1 concentration in the 10 kBq.mL^−1^ exposed groups. Overall, these results suggest a tendency towards a pro-inflammatory response of MS5 cells to ^90^Sr exposure.

## Discussion

Stable strontium is considered as a low toxicity element with a no-observed adverse effect level (NOAEL) in mice of between 40 and 500 mg/kg/day depending on the physiological system studied^11^. In addition, *in vitro* models tested concentrations of 1 × 10^−3^ M stable SrCl_2_ to demonstrate an increase in osteoblastogenesis^13^ and an optimal proliferative stimulation ability of SrCl_2_ on BMSCs within a range of 1 × 10^−3^ to 1 × 10^−5^ M^12^ without any negative effects in these concentration ranges. The concentrated solution of ^90^Sr used in the present study contained 20 μg.ml^−1^ of non-radioactive ^88^Sr as a carrier. For the 10 kBq.ml^−1^ activity, the final concentration in the ^88^Sr culture medium was 2.37 × 10^−9^ M, corresponding to approximately 1 × 10^−4^ fold below the concentrations showing an ability to activate osteoblasts in *in vitro* culture models. As a consequence, we did not use a control with stable ^88^Sr, assuming that the observed effects were solely due to ^90^Sr disintegration and the associated β ray emission. In fact, the mean range of β particles from ^90^Sr in living tissues is in the range of 150–200 μm, and therefore ^90^Sr that accumulates in bone tissue may irradiate the cells closest to the bone tissue. Our work then focused on the induction of DSB and oxidative stress as the primary effects of ionizing radiation on cells lining the bone tissue, i.e. the mesenchymal stromal cells. Results demonstrated that one of the first effects of ^90^Sr exposure, even at low concentrations (1 and 10 kBq.mL^−1^), was the induction of DNA DSB after only 30 minutes of exposure in either primary MSCs or MS5 cell lines.

This peak of γ-H2AX foci was followed by a decrease of γ-H2AX foci frequency down to control values at 24 hours, strongly suggesting the activation of DNA repair mechanisms. This rapid kinetic is similar to that observed in our experiments with external gamma-irradiation at 1 Gy used as positive control, and conforms with other studies focused on external radiation at higher doses^32^ and with a dose-dependent level of DSB induction^33^. The decrease in DSB frequency at 24 hours indicates that the DNA repair process was activated with a similar kinetic as that seen with external irradiation^34^,^35^. This agrees with how the activation of DNA repair mechanisms is independent from the nature of the DNA damaging agent. This rapid repair was confirmed by the increased expression of Rad51, Mre11 and Ku70 at 1 and 24 hours, suggesting implication of both the homologous recombination (HR) and non-homologous end-joining (NHEJ) DNA repair pathways. This rapid DNA repair process was surprising, since the level of exposure and the frequency of DSB were very low, even for the highest ^90^Sr concentration. In fact, the dose rate was estimated to be 6.5 mGy.h^−1^ for the highest ^90^Sr concentration (A. Desbrée, personal communication). However, our results also indicated that the basal level of Ku70, Mre11 and Rad51 proteins was high even in untreated cells, suggesting that in this model cells did not completely reach confluence and were proliferating during exposure. In fact, the appearance of DSB with the progression of replicative forks is a normal physiological mechanism during cell replication, and DNA repair mechanisms are activated during cell division^36^. This may explain the rapid DNA repair seen in our model. However, we did not look at the size of foci at the end of the exposure period, which could have helped to distinguish between persistent foci or foci caused by the constant repair of basal level DSB. In fact, it was shown^37^ that, after external irradiation, the frequency of γ-H2AX foci may return to control levels but with an increased focus size. These enlarged foci are considered as persistent foci with complex DNA damage. Therefore, it cannot be ruled out that in our model the continuous exposure to β ray irradiation might induce persistent foci, with in turn might be responsible for the observed functional changes. At the end of the 7-day exposure period, the level of repair proteins dropped to the basal level. This suggested that a low-dose ^90^Sr exposure is able to induce enough DNA damage to cause the initial activation of DNA repair mechanisms by the MS5, but the basal level of expression for repair proteins is sufficient to manage DSB repair in the long term, thus explaining the rapid disappearance of DSB according to the duration of exposure in our model. Moreover, the simultaneous expression of both Mre11 and Ku70 suggests that, in our model, both HR and NHEJ repair pathways are activated, probably according to the individual cell cycle status and complexation state of chromatin^38^. In general, the rapid repair of DSB during ^90^Sr exposure may contribute to the radiation resistance of MSCs.

One of the mechanisms associated with ionizing radiation is the increase in oxidative stress^20^,^21^. Surprisingly, exposure to ^90^Sr did not induce ROS production. This was confirmed by the stable expression of genes encoding for the main enzymes of the antioxidative system, such as *Cat, MnSOD, Gpx, Gst* and *Gsr*. The enzymatic activity of these enzymes was not tested given the absence of ROS as detected by the H2DCFDA probe. This absence of detectable ROS production is somewhat contradictory with other studies that show increased oxidative stress levels after ionizing radiation^39^, but most of these studies were performed with high doses of irradiation. Moreover, since *in vivo* BMSCs are in a hypoxic environment within the niche^40^, they have a high basal level of anti-oxidative enzymes^41^, which may explain the absence of observed oxidative stress in our model.

One possible consequence of ^90^Sr-induced DNA damage is senescence. Increased expression of genes encoding for *p21* and an increased percentage of cells expressing the protein after 7 days of exposure to ^90^Sr were observed, along with an increase of *p53* phosphorylation, a classical upstream regulator of *p2*^42^,^43^, after 1 hour of contamination. Moreover, the senescent profile of cells is supported by the increase of SA-β-gal activity in cells exposed to ^90^Sr for 7 days. In addition, the induction of senescence by ionizing radiation is known to be a protective mechanism to escape proliferation^22^,^23^. In accordance with this, a significant decrease in cell proliferation was observed in the CFU-F assay after ^90^Sr exposure, although no cell cycle arrest was observed. This result could be due to the non-synchronized proliferation of MS5 cells, thus diluting the signal. It should be noted that the proliferative ability of MS5 cells was also tested using cultures exposed to ^90^Sr during the log phase. The results (not shown) showed a similar decrease in the frequency of CFU-F after exposure to ^90^Sr. This suggests that the induction of senescence is independent of the cell cycle status of the MS5 cells. Senescence is also known to be an alternative mechanism to escape cell death through apoptosis^44^,^45^,^46^. As such, neither cell death nor apoptosis were observed after ^90^Sr exposure in MS5 cells. Similar results were obtained after irradiation of human MSCs up to 20 Gy^47^, thus confirming the radiation resistance of MSCs and the induction of senescence as a protective mechanism.

However, the main point in our study is the demonstration that exposing MS5 cells to ^90^Sr induces several functional changes in BMSCs. One of these changes is the increase in the spontaneous adipocytic differentiation of MS5 cells. MS5 is a pre-adipocytic cell line^29^,^30^ that is no longer able to differentiate towards chondrocytic and osteoblastic lineages. This result appears to contradict other studies showing that radiation-induced senescence is associated with a reduced differentiating ability of bone marrow derived MSCs^48^. On the other hand, a recent study showed an increased differentiation of MSCs toward the adipogenic lineage, in association with a radiation-induced senescent phenotype^49^. A possible explanation for this is the inhibition of the Wnt/β-catenin pathway which is involved in maintaining stem cells in a self-renewing state^50^. This inhibition could take place by activating PPARγ, a major marker of adipocytic differentiation^51^ that is highly expressed in MS5 cells (data not shown).

Another functional change observed in MS5 cell cultures during ^90^Sr exposure was the change in cytokine expression and the resulting change in sustainment of hematopoietic cell regulation. In fact, the increased proliferation of CFU-G strongly suggested a change in cytokine profile of the ^90^Sr-exposed MS5 cells. Although concentrations of the main cytokines implicated in CFC formation (namely SCF, IL-3, IL-6, G-CSF and GM-CSF) were not modified by ^90^Sr exposure, we cannot exclude that the expression of other cytokines implicated in hematopoietic regulation (Flt3-ligand for instance)^52^ could be modified. Accordingly, the extracellular level of anti-inflammatory cytokines (IL-1ra)^53^ and pro-inflammatory cytokines (MCP-1 and TIMP-1)^54^,^55^ changed slightly after 7 days of exposure to ^90^Sr in a way to promote a pro-inflammatory environment by the MS5. This is consistent with the secretory phenotype of senescent cells (senescence associated secretory phenotype, SASP) that present an increased level toward pro-inflammatory cytokines^56^. Nevertheless, these functional changes in MS5 cells remain limited and did not induce changes in LTC-IC proliferation and differentiation. This suggests that the regulation of early HSCs is not modified by ^90^Sr exposure of stromal cells and can also be confirmed by the analysis of SDF-1 concentration. In fact, SDF-1 is strongly involved in the regulation of HSCs^57^ and does not seem to be affected by the exposure to ^90^Sr. This is in accordance with our previous results showing that an *in vivo* chronic exposure to ^90^Sr through ingestion did not induce significant changes in hematopoiesis, although bone physiology was affected^58^.

This *in vitro* study demonstrated for the first time that chronic ^90^Sr exposure at low concentrations is able to induce DNA damage in stromal cells that in turn induce phenotypic and functional changes in these stromal cells, including senescence, differentiation towards the adipocytic lineage, and changes in their ability to support hematopoietic progenitor proliferation. Interestingly, the dosimetric evaluation in our model indicated a dose rate of 6.5 mGy.h^−1^, i.e. 0.3% (after 30 minutes’ exposure) of the 1 Gy external radiation dose used as a positive control. However, ^90^Sr exposure induced a level of DSB close to 10% of the number of DSBs induced by the 1 Gy external exposure. Similar observations were also made for *p53* phosphorylation, *p21* expression and SA-β-Gal expression, while positive controls used in this study clearly demonstrated that the cellular response of MS5 cells to external irradiation was in the normal range compared to other *in vitro* models. This suggests that continuous exposure to ^90^Sr might induce a higher level of cellular damage. This hypothesis may explain the unexpected amplitude of the observed effects compared to the positive controls. These observed effects remains limited in range but such changes in ^90^Sr-exposed BMSCs accumulated in bone tissue may explain some of the previously observed health effects of chronic ^90^Sr ingestion. In fact, we previously observed a change in bone physiology towards bone resorption^9^ and a modification in immune response that could be linked to B cell differentiation in the bone marrow^10^. Additionally, studies in humans suggested decreased hematopoiesis and an altered immune status after internal contamination with ^90^Sr together with a decreased bone remodeling rate^7^,^8^. It is now necessary to verify that chronic ^90^Sr exposure induces similar changes in BMSCs *in vivo*.

## Methods

### Animals and cell culture

Eight-week-old Sprague-Dawley rats and Balb/c mice were purchased from Charles Rivers Laboratories (L’arbresles, France). They received commercial rodent chow and tap water *ad libitum*. The animals were kept at constant room temperature (22 ± 2 °C) with a 12-hour daylight cycle. Mice and rats were anesthetized by injection of a mix of ketamine and xylazine or isoflurane respectively, and blood was harvested by intracardiac puncture. Femurs were then harvested in Modified Eagle’s Medium-α (MEM-α) supplemented with 1% penicillin-streptomycin (PS), 1% L-glutamine and 20% fetal bovine serum (FBS) (all from Invitrogen, Le Pont-de-Claix, France) for subsequent use. All experimental procedures were approved by the animal care committee of the Institut de Radioprotection et de S û reté Nucléaire (IRSN) and conformed to French regulations (Ministry of Agriculture Act No. 87-848, 19 October 1987, modified 29 May 2001).

Rat femurs were flushed using MEM-α supplemented with 10% FBS. After washing and numeration in the presence of acetic acid, bone marrow cells were seeded at 500,000 cells.cm^−2^. 24 hours later, non-adherent cells were washed out and adherent cells were cultured with medium change twice a week until confluence. Rat bone marrow stromal cells (BMSCs) were then used for subsequent experiments in passage 1 or 2. MS5, a murine pre-adipocytic stromal cell line^29^, was cultured in MEM-α supplemented with 1% PS, 1% L-glutamine and 10% FBS. Cells were seeded at a concentration of 5,000 cells.cm^−2^ into 75 cm^2^ flasks and incubated at 37 °C, 5% CO_2_. The medium was changed twice a week until cells reached confluence. Cells were then washed twice with 1x Phosphate Buffer Saline (PBS, Invitrogen) and detached by incubation for 5 minutes at 37 °C with Trypsin-EDTA (Invitrogen). Cells were then harvested, washed twice, counted, and viability assessed by trypan blue exclusion. When indicated, cultures of rat BMSCs or MS5 cells were exposed by adding ^90^SrCl_2_ (CERCA-LEA, Pierrelatte, France) to the culture medium at a final concentration of 1 and 10 kBq.mL^−1^. When required, positive controls were performed and cells were treated either with doxorubicin (2 μM final concentration), rotenone (0.1 μM final concentration, both from Sigma-Aldrich, Saint Quentin Fallavier, France) or γ-irradiated with a ^137^Cs source (0.5 Gy. minute^−1^, IBL, CisBio, Saclay, France).

### CFU-F assay

CFU-F assay was performed as originally described^28^. In summary, cells were seeded into a 25 cm^2^ flask at low concentrations (20, 50, 100 and 200 cells/flask) in triplicate. After 2 weeks of culture at 37 °C and 5% CO_2_, the medium was removed and the cells were washed twice with 1x PBS and fixed 5 minutes with methanol. Cultures were then stained with May-Grünwald Giemsa solutions. Colonies of at least 50 cells were then counted.

### Histological staining

At the end of the exposure period, the medium was removed and frozen at −80 °C for subsequent measurements, and cells were washed twice with 1x PBS. For the staining of adipocytic cells, MS5 cells were fixed 10 minutes at room temperature with 4% paraformaldehyde (PFA), and then washed and incubated for 10 minutes with 2 mL Oil Red O solution (Sigma-Aldrich) in order to stain lipid vacuoles. Cells were then counterstained for 1 minute with 2 mL Mayer’s hemalun and rinsed with tap water. The number of cells containing lipid vacuoles was then assessed in ten fields per well containing at least 50 nuclei. The SA-β-Gal staining was performed following the manufacturer’s instructions (senescence detection kit, Promokine, Heidelberg, Genrmany).

### Immunostaining

For immunostaining of γ-H2AX, after 30 minutes, 24 hours, 72 hours and 7 days of exposure to ^90^SrCl_2_, cells were washed twice with 1x PBS and fixed for 15 minutes at room temperature with a 4% PFA solution. After washing, cells were incubated with lysing solution for 3 minutes at room temperature then with primary rabbit anti γ-H2AX antibody (Millipore, Guyancourt, France) diluted into 2% PBS-BSA (bovine serum albumin) solution for 40 minutes at 37 °C. After washing, cells were incubated for 20 minutes at 37 °C with anti-rabbit IgG coupled to FITC (Life Technologies). After washing, slides were mounted with Vectashield mounting medium containing DAPI (Life Technologies) for nucleus staining. Images were automatically acquired onto fluorescent microscope connected to the Metafer system (Metasystem, Alltussheim, Germany). At least 250 nuclei were captured and the number of γ-H2AX foci/nuclei was assessed using Histolab software (Microvision Instruments, Evry, France).

For *p21* and *p53* detection, cells were stained following the same protocol using a rabbit anti-*p21* (Santa Cruz Biotechnologies, Heidelberg, Germany) or a rabbit anti-*p53* (R&D Systems, Lille, France) and a secondary anti-rabbit IgG coupled to FITC (Life Technologies). Images were acquired on a fluorescent microscope using Histolab software (Microvision Instruments).

### Flow cytometry

Cell cycle analysis was carried out using propidium iodine (PI) staining. In short, after exposure to ^90^SrCl_2_, cells were detached by trypsin incubation, washed twice and counted. 300,000 cells were fixed in 70% ethanol and stored at −20 °C for at least one hour. Cells were then centrifuged 8 minutes at 400 g and the pellet was re-suspended with a sodium citrate solution containing 0.2% Triton X-100, 100 μg.mL^−1^ RNAse and 50 μg.mL^−1^ PI (Sigma) and incubated 30 minutes at 37 °C in the dark. Stained cells were then analyzed on a FACS Canto II (BDIS, Le Pont-de-Claix, France) with the acquisition of at least 10,000 events per condition.

Phenotypic analysis of rat BMSCs was performed using the following directly coupled antibodies: anti-IgG1-FITC, anti-IgG2a-PE, anti-CD44-FITC, anti-CD45-FITC, anti-CD90-PE, anti-CD73-alexa647 (all from BD Pharmingen, Le Pont-de-Claix, France) and anti-CD34-PE (Santa Cruz Biotechnologies). At the first passage, aliquots of 200,000 cells were washed twice in PBS and incubated for 20 minutes in the presence of the indicated antibodies at pre-defined concentrations. Cells were then washed twice and analyzed.

### Intracellular ROS measurement

Intracellular ROS production was detected using the specific dye 2′–7′- dichlorodihydrofluorescein diacetate (H2DCFDA, Sigma-Aldrich), as previously described^59^. Cells were seeded at 2 × 10^6^ cells.mL^−1^ and loaded for 30 minutes in the dark with 20 μM H2DCFDA before exposure to ^90^Sr. After incubation, cells were washed with culture medium and treated with ^90^SrCl_2_ from 15 minutes up to 1 hour. A treatment with 300 μM terbutyl hydroperoxide (tBHP) was used as a positive control. The green DCF fluorescence was analyzed by flow cytometry using the FL1-H wavelength band on 10,000 cells. The results were expressed using mean fluorescent intensity (MFI).

### Senescence associated (SA) β-galactosidase activity measurement

After 7 days of cell culture with ^90^Sr or a senescence inducer (rotenone, 0.1 μM final concentration, Sigma), the medium was removed and replaced by fresh medium containing Bafilomycin A1 at a final concentration of 100 nM (Sigma) and incubated for 1 hour at 37 °C. Cells were then incubated 1 hour at 37 °C with 33 μM 5-Dodeconoylaminofluorescein Di-β-Galactopyranoside (C_12_FDG, Molecular Probes), as previously described^26^,^60^. At the end of the incubation period, cells were harvested as described above and fluorescence was analyzed with a FACSCanto II, with at least 10,000 events recorded for each experimental condition. Results were expressed as mean fluorescence intensity.

### Hematopoietic assays

A colony forming assay was conducted as previously described^58^. In summary, femurs from normal mouse donors were flushed, cells were counted and viability assessed. Cells were then plated at a concentration of 50,000 cells in 1.1 mL of complete methylcellulose medium without cytokines or with erythropoietin (EPO) (both from Stem Cell Technologies, Vancouver, Canada). The methylcellulose medium was previously supplemented with supernatants from MS5 cells cultured in the presence or absence of ^90^SrCl_2._ As a control, supernatant from MS5 cells cultured in the absence of ^90^SrCl_2_ was supplemented with 10 kBq.mL^−1^of ^90^SrCl_2_ to take into account a possible direct effect of ^90^SrCl_2_ on bone marrow progenitor proliferation. Cultures were then incubated at 37 °C, 5% CO_2_ in a humidified atmosphere for 12 days. Colony-forming units-granulocytes (CFU-G) and burst-forming units-erythroid (BFU-E) were scored on day 12 of culture.

Long-term culture initiating-cells (LTC-ICs) were performed using MS5 cell layers as a feeder in 96-well plates as previously described^30^,^31^. At confluence, MS5 cells were exposed for one week to either 1 or 10 kBq.mL^−1^ of ^90^SrCl_2_. Normal mouse bone marrow cells were depleted of mature cells by using lineage (Lin) specific antibody mixture and magnetic beads separation according to the manufacturer’s recommendations (Miltenyi Biotec, Paris, France). The control by FACS analysis showed a population composed of 90.2 ± 7.2% Lin^−^ cells, corresponding to an 8-fold enrichment of Lin^−^ bone marrow cells. After washing the culture layer twice to remove ^90^Sr, culture plates were seeded with 5000, 2500, 1250, 625 or 312 Lin^−^ bone marrow cells in complete long term culture medium (Myelocult, Stem Cell Technologies). A half-medium change was carried out weekly for 5 weeks. Cells were then harvested from each well through trypsin digestion of the stromal layer and plated in complete methylcellulose medium with cytokines (Methocult M3434, Stem Cell Technologies). CFCs were counted on day 12 of culture. The number of positive wells, i.e. containing at least one CFC, was defined and the frequency of LTC-ICs was determined through a linear regression between the number of cells seeded per well and the frequency of negative wells for each culture condition tested.

### Cytokine measurement

Extracellular production of interleukin-3 (IL-3), interleukin-6 (IL-6), interleukin-1 receptor antagonist (IL-1ra), granulocyte colony-stimulating factor (G-CSF), granulocyte-macrophage colony-stimulating factor (GM-CSF), macrophage colony-stimulating factor (M-CSF), stem cell factor (SCF), stromal derived factor-1 (SDF-1), tissue inhibitor of metalloproteinase-1 (TIMP-1) and monocyte chemoattractant protein-1 (MCP-1) were quantified in MS5 supernatant with ELISA kits according to manufacturer’s instructions (R&D Systems, Abingdon, UK). Intracellular levels of cytokines were measured by Mouse Cytokine Array (R&D System) following the manufacturer’s instructions.

### Lactate dehydrogenase measurement

Cytotoxicity of ^90^Sr after one week’s contamination was assessed by extracellular lactate dehydrogenase (LDH) measurement using the Cytotoxicity Detection KitPLUS (Roche Diagnostics, Meylan, France) following the manufacturer’s instructions.

### RT-qPCR

Total mRNA was isolated from cells with the RNeasy mini kit (Qiagen, Courtaboeuf, France) following the manufacturer’s instructions. RNA concentration and integrity were checked by optical density measurement at 230 nm and by 260/280 nm ratio (Thermo Scientific NanoDrop 1000, Labtech, Palaiseau, France). 1 μg of total mRNA was reverse transcribed using the high capacity cDNA reverse transcriptase kit (Applied Biosystems, Courtaboeuf, France) according to the manufacturer’s instructions. Polymerase chain reaction (PCR) assay was performed using the Sybr Green PCR Master Mix (Applied Biosystems) and selected primers (see Table 1) for the measurement of gene expression. Amplification and detection of PCR products were performed using Quant Studio^TM^ 12 K Flex (Life Technologies). The relative expression ratio of each sample was normalized to the geometric mean of the expression level of *Gapdh, Ppia* and *Rpl27* used as housekeeping genes^61^.

### Western blot analysis

Total protein was extracted from cell pellets using a lysing kit (Mammalian Cell Lysing Kit, Sigma) according to the manufacturer’s instructions. Protein concentration was then defined by bicinchoninic acid assay (BCA, Life Technologies). For each sample, 20 μg of proteins were separated by 10% SDS-PAGE and transferred onto nitrocellulose membrane (Invitrogen). The membrane was then incubated for 1 hour with 1x PBS supplemented with 5% fat-free milk and 0.2% Tween 20 to block the non-specific binding sites. Protein immunodetection was performed by incubating the membrane overnight at + 4 °C with polyclonal rabbit anti-Rad51, anti-Ku70 (both from Santa Cruz Biotechnologies) and anti-Mre11 (AbCam). Polyclonal rabbit GAPDH antibody (Santa Cruz Biotechnologies) was used as a loading control. Antibody binding was detected using the corresponding horseradish peroxidase-linked anti-rabbit antibody (AbCam) diluted to 1:5,000 for 1 hour at room temperature and revealed by chemiluminescence on Fujifilm LAS-3000.

### Statistical analysis

Unless otherwise indicated, all cultures were made in triplicate, and all experiments were repeated at least three times. Unless otherwise indicated, all the results are expressed as mean ± standard error of the mean (SEM). Statistical analyses were performed with Sigmaplot software (v11.0, Systat, Paris, France). The comparison between groups was assessed by both two and one-way ANOVA for multiple comparisons and by Student’s t-test for single comparison as indicated. Differences with a p-value less than 0.05 were considered statistically significant.

## Additional Information

**How to cite this article**: Musilli, S. *et al*. DNA damage induced by Strontium-90 exposure at low concentrations in mesenchymal stromal cells: the functional consequences. *Sci. Rep.* **7**, 41580; doi: 10.1038/srep41580 (2017).

**Publisher's note:** Springer Nature remains neutral with regard to jurisdictional claims in published maps and institutional affiliations.

## Supplementary Material

Supplementary Figure 1

## Figures and Tables

**Table 1:** Primers used in this study.

Target gene	Forward primer (3′–5′)	Reverse primer (3′–5′)	Accession number
Lipoprotein lipase (*Lpl*)	AGGCATACAGGTGCAACTCC	GTCCAGTGTCAGCCAGACTT	NM_008509.2
Fatty Acid Binding Protein 4 (*Fabp4*)	TGGAAAGTCGACCACAATAAAGAG	CACCACCAGCTTGTCACCAT	NM_024406.2
Adiponectin (*Adipoq*)	GGATCTGACGACACCAAAAGG	AGGAGAGCTTGCAACAGTAGCAT	NM_009605.4
Glutathione peroxidase (*Gpx*)	GGGACTACACCGAGATGAACG	TCCGCAGGAAGGTAAAGAGC	NM_008160.6
Gluthatione reductase (*Gsr*)	CGGCGATCTCCACAGCAATG	ACCGCTCCACACATCCTGATTG	NM_010344.4
Glutathione-S-transferase (*Gst*)	GACTGTGAGCTGAGTGGAGAAGAA	CCGGCCATTGCAGCAA	NM_008181.3
Manganese Superoxide Dismutase (*MnSOD*)	ACACATTAACGCGCAGATCA	AATATGTCCCCCACCATTGA	NM_001281206.1
Catalase (*Cat*)	AGCGACCAGATGAAGCAGTG	GGGTTACCTCAAAGTATCCAAA	NM_009804.2
Cdk-interacting protein 1 (*p21*)	TTGTCGCTGTCTTGCACTCTGGT	AGACCAATCTGCGCTTGGAGTGAT	NM_007669.4
Transformation-related protein 53 (*p53*)	TTCATTGGGACCATCCTGGC	GGCAGTCATCCAGTCTTCGG	NM_011640.3
Glyceraldehyde-3 phosphate dehydrogenase (*Gapdh*)	AGCTTGTCATCAACGGGAAG	TTTGATGTTAGTGGGGTCTCG	NM_008084
Peptidylprolyl isomerase A (*Ppia*)	GGGTTCCTCCTTTCACAGAA	GATGCCAGGACCTGTATGCT	NM_008907
60 S ribosomal protein L27 (*Rpl27*)	AAGCCGTCATCGTGAAGAACA	CTTGATCTTGGATCGCTTGGC	NM_011289
