# Supplementary material for: DNA damage induced by Strontium-90 exposure at low concentrations in mesenchymal stromal cells: the functional consequences
Source: Sci Rep. 2017 Jan 30;7:41580. doi: 10.1038/srep41580 (PMC5278504; doi:10.1038/srep41580)
Supplement: Supplementary Figure 1 [file srep41580-s1.pdf]

DNA damage induced by Strontium-90 exposure at low concentrations in mesenchymal stromal cells: the functional consequences.

S. Musilli[1], N. Nicolas[1], Z. El Ali [2], P. Orellana-Moreno[1], C. Grand[1], K. Tack[1], S. Kerdine-Römer[2], J.M. Bertho[1]

Supplementary figure 1: Full-size blots uncorrected for contrast corresponding to the blots presented in figure **1d**. **a**: GAPDH detection; **b**: MRE11 detection; **c**: KU70 detection; **d**: RAD51 detection. For GAPDH, MRE11 and KU70, blots were cut in two parts at the level of the 64 KDa molecular weight (MW) marker. The upper part of the blots was used for MRE11 or KU70 detection, while the lower part of the blot was used for GAPDH detection. For RAD51, a full length blot was used. MW markers in **b**, **c** and **d** are registered in visible light and were juxtaposed to the uncorrected chemiluminescence image.

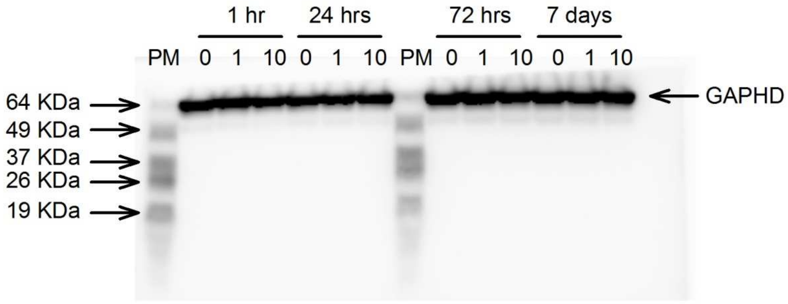
1 hr
24 hrs
72 hrs
7 days
PM 0 1 10 0 1 10 PM 0 1 10 0 1 10
64 KDa
49 KDa
37 KDa
26 KDa
19 KDa
GAPHD

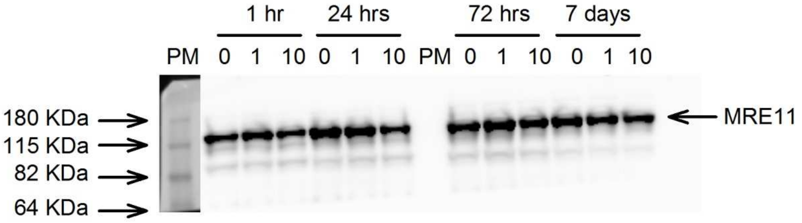
1 hr
24 hrs
72 hrs
7 days
PM 0 1 10 0 1 10 PM 0 1 10 0 1 10
180 KDa
115 KDa
82 KDa
64 KDa
MRE11

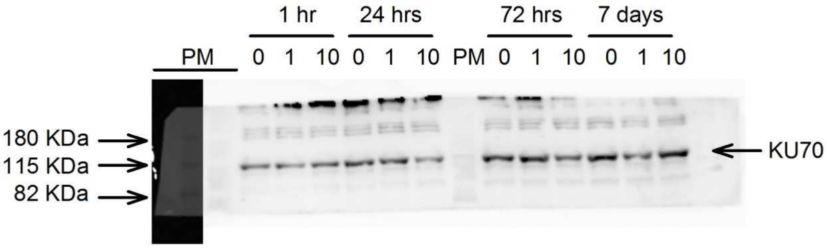
1 hr
24 hrs
72 hrs
7 days
PM 0 1 10 0 1 10 PM 0 1 10 0 1 10
180 KDa
115 KDa
82 KDa
KU70

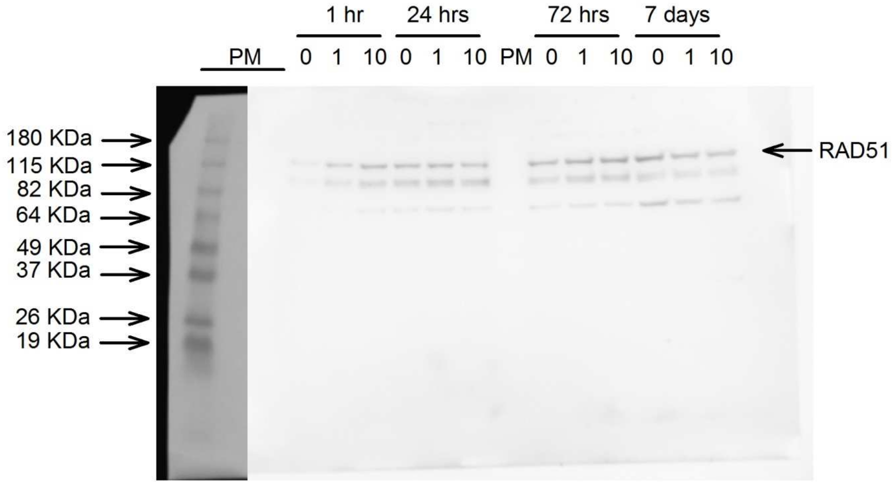
1 hr
24 hrs
72 hrs
7 days
PM 0 1 10 0 1 10 PM 0 1 10 0 1 10
180 KDa
115 KDa
82 KDa
64 KDa
49 KDa
37 KDa
26 KDa
19 KDa
RAD51
